# Facile Histamine Detection by Surface-Enhanced Raman Scattering Using SiO_2_@Au@Ag Alloy Nanoparticles

**DOI:** 10.3390/ijms21114048

**Published:** 2020-06-05

**Authors:** Kim-Hung Huynh, Xuan-Hung Pham, Eunil Hahm, Jaehyun An, Hyung-Mo Kim, Ahla Jo, Bomi Seong, Yoon-Hee Kim, Byung Sung Son, Jaehi Kim, Won-Yeop Rho, Bong-Hyun Jun

**Affiliations:** 1 Department of Bioscience and Biotechnology, Konkuk University, Seoul 143-701, Korea; huynhkimhung82@gmail.com (K.-H.H.); phamricky@gmail.com (X.-H.P.); greenice@konkuk.ac.kr (E.H.); wogus4067@naver.com (J.A.); hmkim0109@konkuk.ac.kr (H.-M.K.); iamara0421@konkuk.ac.kr (A.J.); bom826@naver.com (B.S.); yoonhees@konkuk.ac.kr (Y.-H.K.); imsonbs@konkuk.ac.kr (B.S.S.); susia45@gmail.com (J.K.); 2 School of International Engineering and Science, Jeonbuk National University, 567 Baekje-daero, Deokjin-gu, Jeonju-si, Jeollabuk-do 54896, Korea; rho7272@jbnu.ac.kr

**Keywords:** histamine, fish, gold-silver alloy-embedded silica nanoparticles, surface-enhanced Raman scattering (SERS), reliable and sensitive detection

## Abstract

Histamine intoxication associated with seafood consumption represents a global health problem. The consumption of high concentrations of histamine can cause illnesses ranging from light symptoms, such as a prickling sensation, to death. In this study, gold–silver alloy-embedded silica (SiO_2_@Au@Ag) nanoparticles were created to detect histamine using surface-enhanced Raman scattering (SERS). The optimal histamine SERS signal was measured following incubation with 125 μg/mL of SiO_2_@Au@Ag for 2 h, with a material-to-histamine solution volume ratio of 1:5 and a phosphate-buffered saline-Tween 20 (PBS-T) solvent at pH 7. The SERS intensity of the histamine increased proportionally with the increase in histamine concentration in the range 0.1–0.8 mM, with a limit of detection of 3.698 ppm. Our findings demonstrate the applicability of SERS using nanomaterials for histamine detection. In addition, this study demonstrates that nanoalloys could have a broad application in the future.

## 1. Introduction

Histamine is a common biological substance involved in immune responses, physiological function, and neurotransmission. The consumption of high concentrations of histamine can cause illness ranging from light symptoms, such as a prickling or burning sensation, to serious symptoms, such as erythema, vomiting, diarrhea, headache, angioedema, and urticaria, and even shock or death. Nearly all cases of histamine poisoning are associated with the consumption of fish containing high levels of histidine, which is easily transformed to histamine by decarboxylation if the fish is not correctly stored. Once histamine is produced, it is not easy to completely remove it by heat treatment or freezing. In addition, histamine has no color or odor, which hinders the identification of histamine contamination without noticeable changes in the appearance or smell of the fish [1,2,3,4,5]. According to the European Union (EU) and the U.S. Food and Drug Administration (FDA) standards, the concentration of histamine in fish for consumption must be <100 and 50 ppm, respectively. Therefore, reliable, rapid detection of histamine in fish is essential for food safety and public health, as well as for the global fish industry. Generally, histamine detection is performed using methods such as high-performance liquid chromatography (HPLC), capillary electrophoresis (CE), enzyme linked immunosorbent assay (ELISA), fluorescence quantification, and ion exchange chromatography [6,7,8,9,10,11]. Although these methods are very sensitive, they do have some disadvantages; they use hazardous chemicals and require lengthy pretreatment or specific enzymes, which are expensive and strictly produced. In addition, some protocols indirectly detect histamine via histamine derivatives, which can lead to incorrect results [3,6,7]. 

Surface-enhanced Raman scattering (SERS) is a spectroscopic technique discovered in the 1970s. SERS is an ideal analysis technique, as it can detect single molecules, as well enhance the chemical molecule signal by up to 10^16^-fold. Compared with other analysis methods, SERS requires simple sample preparation and can use a wide range of excitation frequencies, which enables less energetic excitation, resulting in reduced photodamage. Metal colloids, typically silver or gold colloids in suspension or aggregation, have been broadly used for SERS measurements owing to their strong SERS signal and low toxicity [12,13,14]. The useful application of SERS has motivated researchers to develop more reliable SERS techniques. Among those techniques under development, nanoalloy materials have been successfully produced. The abundant variety of metallic alloy compositions, structures, and properties, which can consist of bimetallic nanoclusters (Cu-Ag, Cu-Au, Ag-Au, Ni-Pt, and Fe-Ni) or trimetallic nanoclusters (Cu-Au-Pt, Pd-Ag-Fe, Au-Pt-Ag, and Pd-Au-Pt), has created better stability and synergism that has enabled their widespread application in electronics, engineering, and catalysis [15,16,17,18,19].

Currently, SERS is being increasingly applied in the field of food safety for the detection of harmful substances. Several studies have used silver or gold colloids to detect histamine by SERS [1,2,3,4,5]. Compared with other standard methods, such as HPLC, these SERS methods have the advantages of sensitivity, reliability, and easy fabrication; however, precise control of the size and amount of the aggregated particle clusters is difficult because of the heterogeneous formation of the metal particles. The use of a template, such as silica particles or polymer beads, to accumulate silver or gold nanoparticles (NPs) has been widely established in order to control particle size [20,21,22,23,24,25,26,27,28]. Recently, our group produced an Au-Ag alloy on silica nanoparticles as a highly sensitive and reliable SERS probe that can detect molecular targets at very low concentrations [29,30,31,32,33,34,35,36,37,38,39,40,41,42]. Based on these studies, we investigated histamine detection using an Au-Ag alloy on silica particles as a material for SERS.

## 2. Results and Discussion

### 2.1. Characterization of the SiO_2_@Au@Ag NPs

The SiO_2_@Au@Ag NP material was prepared based on studies conducted by the Pham group revealing that SiO_2_@Au@Ag NPs exhibit a high Raman enhancement effect [39,43,44]. Silica NPs were produced using the Stöber method. Subsequently, the surfaces of the silica NPs were covered with Au NPs on which an Ag shell was created. 

**Figure 1:**
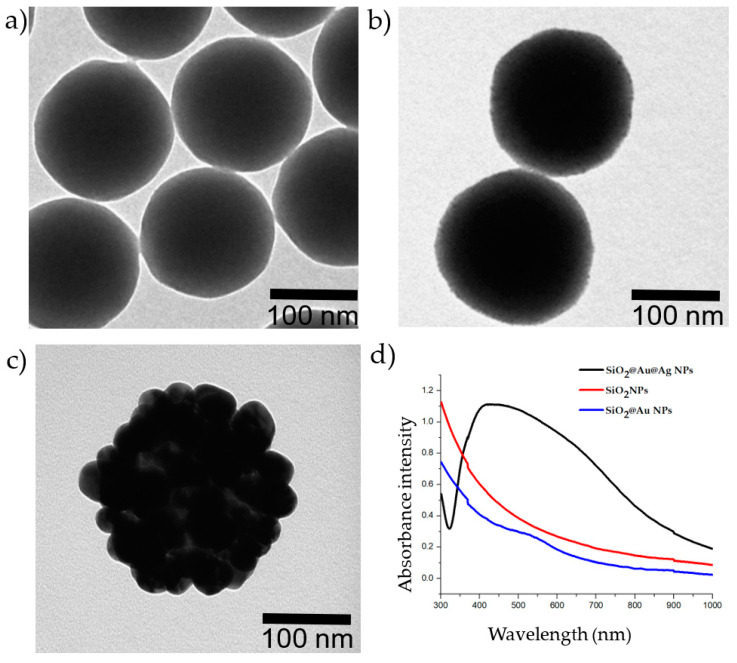
Images of the nanoparticles and UV–Vis absorbance of the nanoparticles. (**a**) Transmission electron microscopy (TEM) image of silica (SiO_2_) NPs; (**b**) TEM image of SiO_2_@Au NPs; (**c**) TEM image of SiO_2_@Au@Ag NPs; (**d**) UV–Vis absorbance of NPs. Red: 1000 µg/mL SiO_2_ NPs; blue: 250 µg/mL SiO_2_@Au NPs; black: 20 µg/mL SiO_2_@Au@Ag NPs.

Figure 1 shows transmission electron microscopy (TEM) images of the nanomaterials. The average diameter of the SiO_2_ NPs was 160 nm (1a). SiO_2_ NPs covered by Au NPs (2–3 nm) are shown in Figure 1b. The surface of the SiO_2_@Au NPs was thoroughly coated with an Ag shell (1c), with clear nanogaps between the Ag NPs, which will provide the best Raman signal [39]. As shown in Figure 1d, while the SiO_2_ suspension did not exhibit UV–Vis absorbance in the 300–1000 nm range, the SiO_2_@Au NP colloid showed a peak at approximately 520 nm. Once the Ag NPs were embedded onto SiO_2_@Au, the absorbance of the SiO_2_@Au@Ag suspension showed a wide band from 320 to nearly 800 nm.

### 2.2. Optimization of Histamine Detection

As the SERS signal is affected by many factors, we sought to determine the effect of target volume, incubation time, solvent pH, and material concentration on SERS signal. The SERS spectra of histamine-modified SiO_2_@Au@Ag were observed at 850, 1001, 1200, 1258, 1263, 1318, 1353, 1449, 1536, 1603, and 1641 cm^−1^ (Figure S1 and Table S1, Supplementary Materials). The bands at 1641, 1603, 1536, 1353, and 850 cm^−1^ were assigned to ring stretching; the bands at 1258 and 1001 cm^−1^ were assigned to ring bending; the band at 1449 cm^−1^ was assigned to the bending of the CH_2_ side chain; the band at 1318 cm^−1^ was assigned to CH_2_ wagging; and the bands at 1200 and 1263 cm^−1^ were assigned to ring breathing [45,46,47,48,49,50]. For simple evaluation, we considered an intensity of wavelength number of 1603 cm^−1^, which might be due to ring stretching [45,46,47,48,49,50], as the highest histamine Raman shift peak.

#### 2.2.1. Effect of Target Volume on Histamine Detection

**Figure 2:**
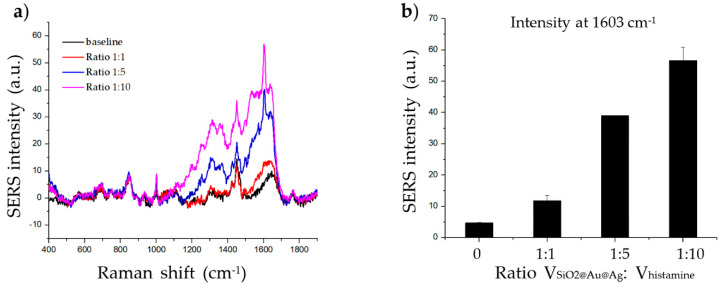
Effect of target volume on histamine detection. (**a**) Raman signal of histamine incubated with SiO_2_@Au@Ag nanoparticles (NPs) at three volume ratios (1:1, 1:5, and 1:10) after 2 h. (**b**) The Raman intensity of histamine incubated with SiO_2_@Au@Ag NPs at various volume ratios after 2 h (at 1603 cm^−1^).

As the SERS signal is affected by the amount of target molecule on the surface of the material, we carried out an experiment in which we incubated 20 µg of SiO_2_@Au@Ag NPs (100 µL) with different volumes of 1 mM histamine (100, 500 µL, and 1000 µL); the mean ratio between the volume of the material and histamine was 1:1, 1:5, and 1:10, respectively. As shown in Figure 2, the SERS signal increased with increasing volume, as the amount of histamine absorbed onto the surface of the material increased. Therefore, the SERS signal at a 1:5 and 1:10 ratio was clearer than that at a 1:1 ratio. The 1:5 ratio was chosen for subsequent experiments.

#### 2.2.2. Effect of Incubation Time on Histamine Detection

**Figure 3:**
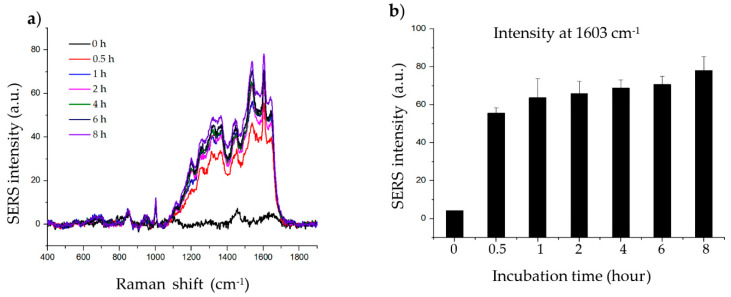
Effect of incubation time on histamine detection. (**a**) Raman signal of histamine incubated with SiO_2_@Au@Ag nanoparticles (NPs) after 0.5, 1, 2, 4, 6, and 8 h. (**b**) Raman intensity of histamine incubated with SiO_2_@Au@Ag NPs after 0.5, 1, 2, 4, 6, and 8 h (at 1603 cm^−1^).

The incubation step allows the target molecule to adsorb onto the surface of the material. To determine the effect of histamine incubation time, histamine was incubated with 20 µg of material for 0, 0.5, 1, 2, 4, 6, and 8 h. As shown in Figure 3, the intensity of the SERS signal increased up to 1 h of incubation. After 1 h, the SERS signal of the histamine gradually increased with further incubation. The signals at wave number 1603 cm^−1^ are clear enough irrespective of experimental incubation time; thus, 2 h of incubation was chosen for subsequent experiments as the intensity at 2 h represents approximately the average of the intensity obtained after incubation for the other time periods.

#### 2.2.3. Effect of Solvent pH on Histamine Detection

**Figure 4:**
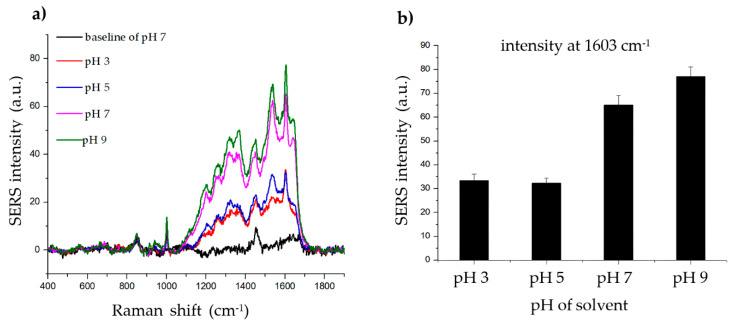
Effect of solvent pH on histamine detection. (**a**) Raman signal of histamine incubated with SiO_2_@Au@Ag nanoparticles (NPs) after 2 h in solvents (phosphate-buffered saline-Tween 20 (PBS-T)) with different pH values (3, 5, 7, and 9). (**b**) The Raman intensity of histamine in solvents (PBS-T) with different pH values (3, 5, 7, and 9) (at 1603 cm^−1^).

To determine the effect of pH on the SERS signal of the histamine, phosphate-buffered saline-Tween 20 (PBS-T) solvents with various pH values (3, 5, 7, and 9) were created by adjusting the pH with hydrochloric acid (HCl) and sodium hydroxide (NaOH). As shown in Figure 4, the SERS signal of the histamine was strong and clear at all four pH values. However, based on this result, the binding between the histamine and the Ag shell appears to be better in an alkaline environment. Thus, pH 7 was chosen for subsequent experiments as it is near the physiological pH.

#### 2.2.4. Effect of the Material Concentration on the SERS Signal of Histamine

**Figure 5:**
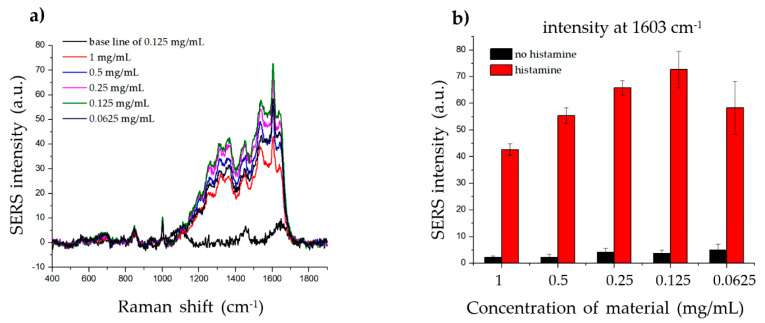
Effect of material concentration on histamine detection. (**a**) The Raman signal of the same concentration of histamine incubated with 1, 0.5, 0.25, 0.125, and 0.0625 mg/mL of SiO_2_@Au@Ag nanoparticles (NPs). (**b**) The Raman intensity of histamine for different concentrations of SiO_2_@Au@Ag NPs (at 1603 cm^−1^).

To determine the effect of the material concentration on the SERS signal of histamine, we incubated the same amount of histamine with different concentrations of material (1, 0.5, 0.25, 0.125, and 0.0625 mg/mL) and measured the Raman signal. As shown in Figure 5, the strongest SERS signal was detected when 0.125 mg/mL material was incubated with histamine, while weaker SERS signals were detected at both higher and lower concentrations. These results indicate that the dispersion density of histamine on the surface of the material significantly affected the SERS signal. Although the high and low concentrations of the material did not generate a sufficiently robust SERS signal, any of the concentrations can be used, as the intensities at 1603 cm^−1^ were strong and could be clearly observed.

### 2.3. The Limit of Detection (LOD) of Histamine

**Figure 6:**
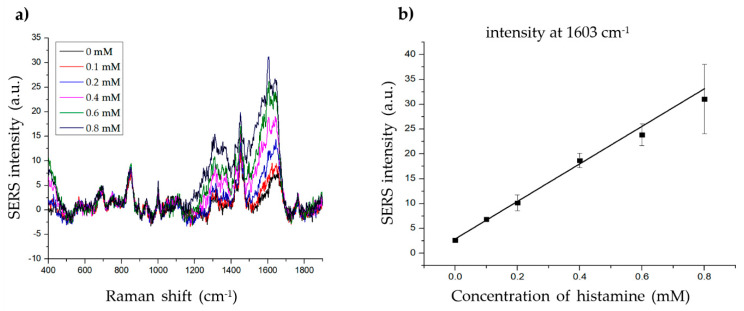
Determining the limit of detection (LOD) of histamine. (**a**) The Raman signal of histamine at different concentrations (0, 0.1, 0.2, 0.4, 0.6, and 0.8 mM). (**b**) The standard linear plot of histamine concentration vs. SERS intensity at 1603 cm^−1^.

To determine the LOD of histamine, we measured the SERS signal at various concentrations of histamine (0.1–0.8 mM) with 20 µg of material (Figure 6a). The intensity at 1603 cm^−1^ increased proportionally with increasing histamine concentration (Figure 6b). The linear calibration formula was determined as y = 37.79951x + 2.89144, R^2^ = 0.99081 (x = histamine concentration, y = SERS intensity at 1603 cm^−1^). The LOD of histamine was 0.033 mM (3.698 ppm) with a signal-to-noise ratio (S/N) = 3, which is considerably lower than the standards described by the FDA (50 ppm) or EU (100 ppm). The LOD of the present method (3.698 ppm) was also comparable to that of existing histamine detection methods such as ELISA (1–17 ppm) [6,8,11], HPLC (0.1–25 ppm) [7,8,10,51], and SERS (5–15 ppm) [2,4,5]. Although the LOD of the present method was not lower than the lowest ELISA and HPLC LODs, it remains useful, as its LOD is lower than the highest LOD values of the other methods. Furthermore, SERS-based methods, including the present method, are suitable for biological applications owing to several advantages, such as low cost, high efficacy, fewer harmful chemicals, non-destructive features, and simple sample preparation. The present method also showed a lower LOD than previous SERS-based histamine detection methods (3.986 vs. 5–15 ppm), owing to the use of Au-Ag alloy NPs instead of Au or Ag NPs. Thus, these results indicate the possible application of this method for histamine detection in fish samples. Additionally, these results also demonstrate a novel SERS-based method using gold-silver alloy-embedded silica NPs for molecular determination. 

## 3. Materials and Methods 

### 3.1. Chemicals and Materials

All reagents were used as received from the suppliers without further purification. Tetraethylorthosilicate (TEOS), 3-aminopropyltriethoxysilane (APTS), polyvinylpyrrolidone (PVP) (Mw 40,000), silver nitrate (AgNO_3_), L-ascorbic acid, Tween 20, tetrakis(hydroxymethyl)phosphonium chloride (THPC), gold (III) chloride trihydrate (HAuCl_4_), and histamine dihydrochloride were purchased from Sigma-Aldrich (St. Louis, MO, USA). Ethyl alcohol (EtOH) and aqueous ammonium hydroxide (NH_4_OH) were purchased from Daejung (Siheung, South Korea). HCl and NaOH were purchased from Samchun (Pyeongtaek, South Korea). Phosphate-buffered saline (PBS; 20×) was purchased from Dyne Bio (Seongnam, South Korea). Ultrapure water (resistivity 18.2 MΩ×cm) was produced using a Millipore water purification system (EXL water purification, Vivagen Co., Ltd., Seongnam, South Korea).

### 3.2. Preparation of SiO_2_@Au@Ag NPs

The SiO_2_@Au@Ag NP material was prepared using silica NPs produced via the Stöber method, with an average diameter of approximately 160 nm. Following amine-functionalization performed by incubating a mixture containing 200 mg of silica NPs, 4 mL of absolute EtOH, 200 µL of APTS, and 40 µL of NH_4_OH for 12 h, the silica NPs were incubated with Au NPs (2–3 nm) prepared by reducing HAuCl_4_ with THPC for 12 h with gentle shaking at 25 °C. The surfaces of the aminated silica NPs were covered with Au NPs. An Ag shell was created on the surface of the SiO_2_@Au NPs by reducing AgNO_3_ in the presence of ascorbic acid and PVP; 200 µL of 200 µg/mL SiO_2_@Au@Ag NPs were well dispersed in 9.8 mL of 1 mg/mL PVP solvent and then 20 µL of 10 mM AgNO_3_ was added, followed by the addition of 20 µL of 10 mM ascorbic acid. This suspension was slowly stirred for 15 min for the reduction of Ag^+^ ions to Ag. The reaction was repeated to obtain an AgNO_3_ concentration of 300 µM. The SiO_2_@Au@Ag NPs were collected by centrifugation at 8500 rpm for 15 min. Following several washes with EtOH to remove excess reagent, the SiO_2_@Au@Ag NPs were re-dispersed in absolute EtOH to obtain a 200 µg/mL SiO_2_@Au@Ag NP solution. 

### 3.3. Histamine Detection

The histamine solution was prepared by dissolving histamine dihydrochloride in PBS-Tween 20 (1%; PBS-T), pH 7. To absorb histamine on the surface of the SiO_2_@Au@Ag NPs, 100 µL of a 1 mM histamine solution were incubated with 100 µL of a 200 µg/mL SiO_2_@Au@Ag NP suspension for 2 h, followed by centrifugation for 15 min at 11,000 rpm to collect the colloids. The NPs was washed several times with PBS-T (pH 7) to remove excess reagent. The SiO_2_@Au@Ag@Histamine NPs were re-dispersed in 100 μL of PBS-T (pH 7) to obtain a 200 µg/mL SiO_2_@Au@Ag@Histamine NP suspension. For optimization, each condition, including incubation time, solvent pH, and volume of histamine solution, was changed. The LOD of histamine was determined by varying the concentration of histamine. The control sample (baseline) consisted of only SiO_2_@Au@Ag NP material in PBS-T (pH 7) solvent. Each experiment was conducted three times.

### 3.4. SERS Measurement of SiO_2_@Au@Ag@Histamine

The SERS signals were measured using a DXR 2 Raman Microscope System (Thermo Fisher Scientific, Waltham, MA, USA) with a 532-nm laser excitation source and 10× objective lens. Liquid samples were measured in a capillary tube with a laser power of excitation of 10 mW for 5 s. The size of the laser beam spot was approximately 2.0 µm and the sites were randomly selected. The SERS spectra were collected in the 400–1900 cm^−1^ wavenumber range. Each sample was measured three times. The highest peak at wave number 1603 cm^−1^ was selected for analysis.

## 4. Conclusions

In this study, histamine was successfully detected by SERS using a SiO_2_@Au@Ag alloy nanomaterial. The best SERS signal was obtained using an incubation time of 2 h, a material-to-histamine solution volume ratio of 1:5, PBS-T solvent at pH 7, and material concentration of 0.125 mg/mL; using this protocol, the LOD of histamine was 3.698 ppm. To the best of our knowledge, this study is the first to report histamine detection using gold-silver alloy-embedded silica nanoparticles and provides the basis for further research that could be applied to the detection of histamine in real samples. In addition, this study demonstrates that nanoalloys are novel materials that could have a broad application in the future.

## Supplementary Materials

Supplementary materials can be found at https://www.mdpi.com/1422-0067/21/11/4048/s1.

## Abbreviations

AgNO_3_	Silver nitrate
APTS	3-Aminopropyltriethoxysilane
CE	Capillary electrophoresis
ELISA	Enzyme linked immunosorbent assay
EtOH	Ethyl alcohol
EU	European Union
FDA	U.S. Food and Drug Administration
HAuCl_4_	Gold (III) chloride trihydrate
HCl	Hydrochloric acid
HPLC	High-performance liquid chromatography
LOD	Limit of detection
NaOH	Sodium hydroxide
NH_4_OH	Ammonium hydroxide
NPs	Nanoparticles
PBS-T	Phosphate-buffered saline-Tween 20
PVP	Polyvinylpyrrolidone
S/N	Signal to noise ratio
SERS	Surface-enhanced Raman scattering
SiO_2_@Au@Ag	Gold–silver alloy-embedded silica
TEM	Transmission electron microscopy
TEOS	Tetraethylorthosilicate
THPC	Tetrakis(hydroxymethyl)phosphonium chloride
