# Supplementary material for: Facile Histamine Detection by Surface-Enhanced Raman Scattering Using SiO2@Au@Ag Alloy Nanoparticles
Source: Int J Mol Sci. 2020 Jun 5;21(11):4048. doi: 10.3390/ijms21114048 (PMC7311956; doi:10.3390/ijms21114048)
Supplement: Supplementary file 1 [file ijms-21-04048-s001.pdf]

Facile Histamine Detection by Surface-Enhanced Raman Scattering using SiO_2@Au@Ag Alloy Nanoparticles

Kim-Hung Huynh [1], Xuan-Hung Pham [1], Eunil Halm [1], Jaehyun An [1], Hyung-Mo Kim [1], Ahla Jo [1], Bomi Seong [1], Yoon-Hee Kim [1], Byung Sung Son [1], Jaehi Kim [1], Won-Yeop Rho [2] and Bong-Hyun Jun [1,*]

[1] Department of Bioscience and Biotechnology, Konkuk University, Seoul 143-701, Korea. K.-H.H.; huynhkimhung82@gmail.com, X.-H.P.; phamricky@gmail.com, E.H. greenice@konkuk.ac.kr, J.A.; wogus4067@naver.com, H.-M.K.; hmkim0109@konkuk.ac.kr, A.J.; iamara0421@konkuk.ac.kr, B.S.; bom826@naver.com, Y.-H.K.; yoonhees@konkuk.ac.kr, B.S.S.; imsonbs@konkuk.ac.kr, B.-H.J.; J.K. susia45@gmail.com; B.-H.J. bjun@konkuk.ac.kr

[2] Chonbuk Natl Univ, Sch Int Engn & Sci, 567 Baekje Daero, Jeonju Si 54896, Jeollabuk Do, Korea. W.-Y. R.; rho7272@jbnu.ac.kr

Name of Corresponding Author: Bong-Hyun Jun, Ph.D.

Tel.: +82-2-450-0521, Fax: +82-2-3437-1977, E-mail: bjun@konkuk.ac.kr (B.-H. Jun)

Keywords: histamine; fish; gold-silver alloy-embedded silica nanoparticles; surface-enhanced Raman scattering (SERS); reliable and sensitive detection.

Assignment of SERS Histamine Bands

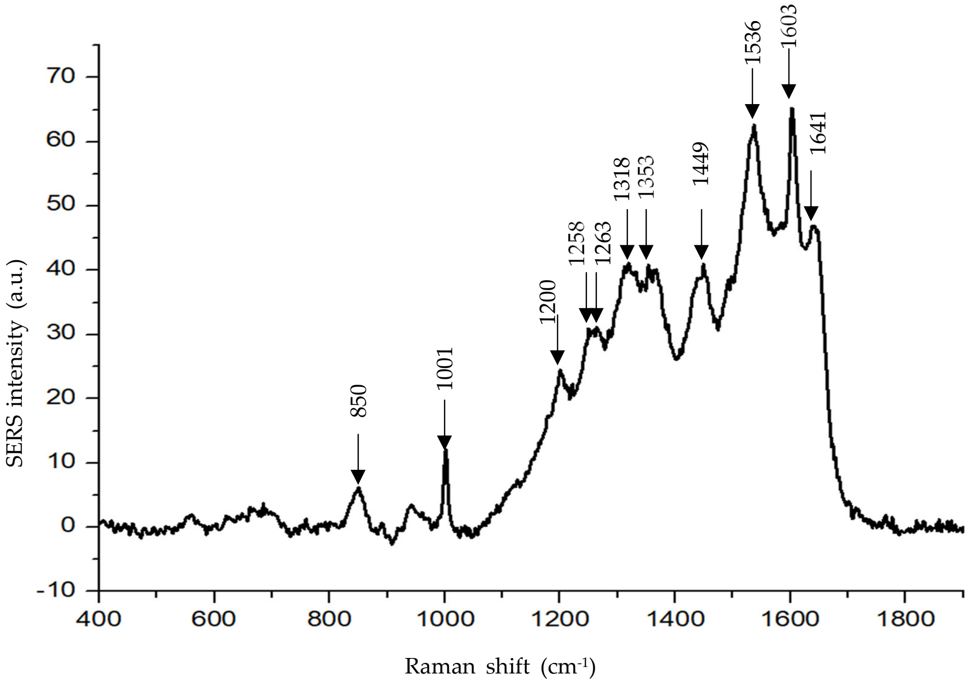

Figure S1: SERS spectra of histamine absorbed on the surface of SiO_2@Au@Ag.

Table S1: Wave number (cm^{-1}) and assignment of Raman spectra of dissolved histamine

Experiment	B3LYP/6-311G neutral histamine (calculated)	Histamine in D_2O	Histamine bands using Ag NPs made by citrate	Histamine in pH 7.4	Assignment
	Ref [49]	Ref [45]	Ref [48]	Ref [46]	
1641				1637	Stretching of ring
1603	1607	1604			Stretching of ring
1536			1533		Stretching of ring
1449	1448	1446		1448	Bending of side chain (CH_2)
1353	1353	1356	1353		Stretching of ring
1318	1313	1321	1320		Wagging of CH_2
1263			1268		Ring breathing
1258	1251	1254			Bending of ring (CH)
1200	1200	1211		1194	Ring breathing
1001	998	1004	996		Bending of C-H in ring
850				852	Stretching of C-C
